# Achieving *Remission* in Chronic Rhinosinusitis With Nasal Polyps Depends on Practical Utilization of Current Criteria and Does not Reflect Therapy *Response*: Results From a Multi‐National Cohort

**DOI:** 10.1002/clt2.70211

**Published:** 2026-10-04

**Authors:** Jan Hagemann, Tobias Albrecht, Sanna Toppila‐Salmi, Marie Lundberg, Annina Lyly, Katharina Geißler, Friederike Bärhold, Caroline Beutner, Inga Nierkamp, Mandy Cuevas, Nadine Gunder, Adam Chaker, Moritz Gröger, Ludger Klimek, Sven Becker

**Affiliations:** ^1^ Center for Rhinology and Allergology Wiesbaden Germany; ^2^ Department of Otorhinolaryngology, Head and Neck Surgery Universitätsmedizin Mainz Mainz Germany; ^3^ German National CRS‐Registry (“CRS‐Register”) Kopf‐Hals‐Chirurgie Ärzteverband Deutscher Allergologen e.V. & Deutsche Gesellschaft für Hals‐Nasen‐Ohrenheilkunde Wiesbaden Germany; ^4^ Department of Otorhinolaryngology Head and Neck Surgery University Medical Center of Eberhard Karls University Tübingen Germany; ^5^ Department of Otorhinolaryngology University of Eastern Finland Joensuu and Kuopio Finland; ^6^ Wellbeing Services County of Pohjois Savo Kuopio Finland; ^7^ Department of Allergology Inflammation Center Helsinki University Hospital and University of Helsinki Helsinki Finland; ^8^ Department of Otorhinolaryngology Helsinki University Hospital and University of Helsinki Helsinki Finland; ^9^ Department of Otorhinolaryngology Jena University Hospital Thuringia Germany; ^10^ Department of Dermatology Venereology and Allergology University Medical Center Göttingen Göttingen Germany; ^11^ Department of Otorhinolaryngology University Clinic of Munster Münster Germany; ^12^ Department of Otolaryngology Head and Neck Surgery University Hospital Carl Gustav Carus TUD Dresden University of Technology Dresden Germany; ^13^ Deptartment of Otorhinolaryngology and Center for Allergy and Environment (ZAUM) TUM School of Medicine Klinikum rechts der Isar Technical University of Munich Munich Germany; ^14^ Department of Otorhinolaryngology, Head and Neck Surgery Ludwig‐Maximilian‐University Munich Munich Germany

**Keywords:** asthma, biologics, nasal polyps, paranasal sinus diseases, *Remission*

**Abbreviations**
CRSwNP: chronic rhinosinusitis with nasal polyps
IgE: Immunglobulin E
IL‐4R: Interleukin‐4‐receptor
IL‐5: Interleukin‐5
TSLP: thymic stromal lymphopoietin

## Author Contributions

**Jan Hagemann:** conceptualization, investigation, writing – original draft, writing – review and editing, methodology, formal analysis, data curation, project administration, visualization, validation, supervision, resources. **Tobias Albrecht:** investigation, conceptualization, writing – review and editing, supervision, methodology. **Sanna Toppila‐Salmi:** conceptualization, writing – review and editing, investigation, supervision, validation, methodology, formal analysis. **Marie Lundberg:** conceptualization, investigation, methodology, supervision, writing – review and editing, formal analysis. **Annina Lyly:** formal analysis, methodology, writing – review and editing, investigation. **Katharina Geißler:** investigation, methodology, formal analysis, writing – review and editing. **Friederike Bärhold:** visualization, methodology, writing – original draft, formal analysis. **Caroline Beutner:** methodology, investigation, supervision, writing – review and editing, formal analysis, conceptualization. **Inga Nierkamp:** investigation, methodology, formal analysis, writing – review and editing. **Mandy Cuevas:** investigation, methodology, writing – review and editing. **Nadine Gunder:** investigation, formal analysis, methodology, writing – review and editing. **Adam Chaker:** writing – review and editing, investigation, supervision, methodology. **Moritz Gröger:** investigation, methodology, writing – review and editing, supervision. **Ludger Klimek:** supervision, project administration, methodology, writing – review and editing. **Sven Becker:** writing – review and editing, methodology, formal analysis, investigation, validation.

## Funding

The authors have nothing to report.

## Conflicts of Interest

J.H. declares consulting fees from HAL Allergy, Sanofi Genzyme D GmbH, GlaxoSmithKline (GSK) D GmbH, GSK Global, Novartis Pharma D GmbH, LETI Pharma, Stallergenes, Bencard, outside the here‐submitted work. M.G. declares consulting fees from Sanofi, GlaxoSmithKline (GSK), AstraZeneca, and Novartis. A.L. reports consultancies for ALK, AstraZeneca, GSK, MSD, Orion Pharma and Finnish Lung Health Association. A.L. was an employee of AstraZeneca, outside of the submitted work. S.T.‐S. reports consultancies for ALK‐Abelló, AstraZeneca, Clario, ERT, GlaxoSmithKline, Novartis, Sanofi Pharma, OrionPharma, Roche Products and, grants from GlaxoSmithKline and Sanofi, outside of the submitted work. M.C. received personal fees for lectures from ALK‐Abelló, Allergopharma, AstraZeneca, Bencard Allergy Therapeutics, Celltrion, GlaxoSmithKline (GSK), HAL Allergy, LETI Pharma, Novartis, Roxall, Sanofi‐Aventis, and Stallergenes NeilMed, outside the here submitted work. Other non‐financial interests of authors: German Society of Allergology (AeDA), German Society of Otorhinolaryngology, Head and Neck Surgery, European Academy of Allergy and Immunology. All other authors declare no conflicts of interest. This study has not been funded by any public or private institution or third party. Data is available upon request to the corresponding author. J.H., M.L. and S.T.‐S. conceptualized the study. JH drafted the manuscript. J.H., M.L., A.L. and S.T.‐S. performed statistical analyses. All authors critically revised, edited and commented the manuscript. All authors gave permission to submission and contributed equally to the revision process.

Dear Editor,

Recent advances in biologic therapy for severe chronic rhinosinusitis with nasal polyps (CRSwNP), asthma, and other type‐2 inflammatory diseases have raised attention to patient‐reported outcomes. Long and sustained effects of biologic drugs targeting IgE, TSLP, IL‐4R, and IL‐5 signaling for CRS and/or asthma have created a situation where *Remission* and other controlled states are observable long‐term. However, well‐defined and meaningful diagnostic biomarkers predicting therapeutic *Response* are yet to be found. Furthermore, *Remission* and *Control* states require unambiguous definitions to enable structured communication between physicians and patients [1]. In a pivotal effort, an expert consensus of renowned specialists in ENT, pulmonology, allergology, and immunology has suggested criteria of how to define disease states such as *Control* and *Remission* and therapeutic goals in CRSwNP, omitting physician‐reported parameters for the definition of *Control* and emphasizing persistent absence of burdensome symptoms for patients [2].

In an orchestrated and multi‐national effort applying these recently suggested and novel criteria to a large cohort of biologic drug applications (*n* = 369), we identified three main findings: (1) Disease *Remission*, defined as sustained *Control* for > 12 months in absence of rescue treatments and active disease, was achieved only in a minority of cases, despite partial or full *Response* to biologic therapy; (2) under biologic drugs, disease *Control* and *Remission* are therapeutic goals possibly facilitated in the presence of co‐morbidities such as comorbid asthma and salicylate intolerance (N‐ERD), and (3) the practical application of current definitions for disease *Control* and *Remission* is unfolding substantial challenges with regards to cut‐offs, inter‐observer variability, and choice of reporting method. Furthermore, we feel that these definitions could be further improved by an orchestrated and data‐driven approach to compensate for these challenges. In addition, incorporating the status of known co‐morbidities and possible side‐effects of the therapy in use might be important with regards to *Remission* as a therapeutic goal.

Rhinologic centers in Germany and Finland assessed *Control* and *Remission* in *n* = 369 patients with severe CRSwNP (mean age 55.2 years), treated with Dupilumab (*n* = 274), Mepolizumab (*n* = 72), or Omalizumab (*n* = 29) for up to 36 months (total *n* = 88 after 3 years observation, Supporting Information S1: Figure 1). Six patients switched biologic drugs (all to Dupilumab) during the observation period but have been observed more than 12 months for each individual course of biologic. The study respected the Declaration of Helsinki and Ethics approval was given to each center separately under local regulations for anonymous, retrospective analyses. As suggested by previous literature [3], *Control* was defined based on patient‐reported outcomes such as visual analogue scales (VAS) and sinonasal outcome test of 22 predefined items (SNOT‐22), *Remission* was defined on sustained disease *Control* for all available assessment spanning > 12 months in addition to HCP‐reported endoscopy findings (performed locally by treating rhinologist) and absence of rescue treatments. EUFOREA suggest “absence of active disease” as decisive for the goal of *Remission*; this is most commonly interpreted as an NPS of =< 2 (1 per side), even though this leaves room for an ongoing scientific discussion if edema and secretions find room in the future assessment of *Remission*. Heterogeneity of data collection parameters is unavoidable to date in multi‐center collaborative approaches and represents a major challenge yet to overcome in the future. VAS‐based symptom data was available for *n* = 179 cases at 12 months. Using these data sets with VAS information only, disease *Control* was achieved in roughly 30% of cases already at 6 months, staying stable over the next 2.5 years with minor variations (Figure 1A). Whenever VAS‐based symptom data was not available, “adapted” criteria for *Control* were put into place in *n* = 190 cases at 12 months (VAS global symptoms < 5 from 0 to 10 cm and SNOT‐22 < 25 points and Sniffin’Sticks 12 item identification testing > 8). The use of adapted criteria assessing *Control* and *Remission* was leading to higher rates of symptom control, compared to VAS‐based criteria alone (38.0 vs. 44.7% at 12 months, *p* = 0.11). This difference was not statistically different, but should be analyzed in further detail: most commonly there was a discrepancy between self‐reported olfactory function and psychophysical olfactory testing results (12‐item Sniffin’Sticks identification test), which has been a known phenomenon in the past [4]. Merging all data sets, comprising three different biologic therapies, disease *Control* was observed in 36.3% at 6 months, rising to 46.5% at 24 months, and stabilizing above 40% at 36 months (Figure 1A). When stratified in sub‐groups for known co‐morbidities “asthma” and “N‐ERD”, the rates of *Control* clearly rose to up to 50% for asthma and up to 64.3% for N‐ERD during the observation period (Figure 1B) when strictly focusing on VAS‐based reporting of symptomsRates were higher with merged datasets of VAS‐based and adapted criteria (61.8% and 79.1%, respectively; data not shown).

**FIGURE 1:**
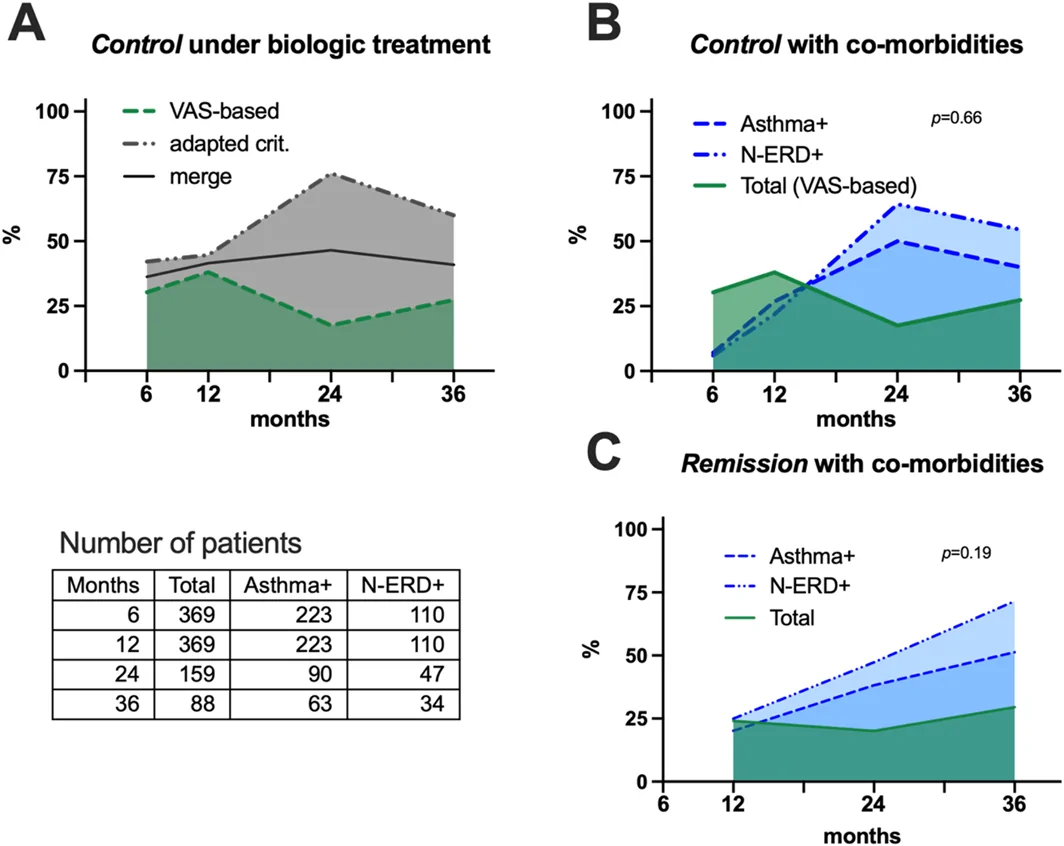
Disease states of *control* and *Remission* after 12–36 months of biologic therapy in CRSwNP patients. (A and B): *Control* was assessed according to Fokkens et al. [2] both purely VAS‐based approach (VAS nasal obstruction, VAS olfactory impairment < 5 from 0 to 10 cm) and based on adapted criteria (VAS global symptoms < 5 from 0 to 10 cm and SNOT‐22 < 25 points and Sniffin’Sticks 12 item identification testing > 8) [3]. (C) *Remission* was rewarded based on sustained *control* for the past 12 months in absence of surgical or steroid rescue intervention and nasal polyp scoring (nasal endoscopy) of ≤ 1 per side (merged control data sets shown). Statistical analysis by Repeated‐measures one‐way ANOVA on GraphPad Prism Version 10. (A) Control under biologic treatment (dupilumab, mepolizumab, omalizumab). (B and C) Sub‐analysis of *control* and *Remission* depending on presence of asthma and/or N‐ERD as comorbidities.

Disease *Remission*, on the other hand, occurred in 24.1% after 12 months and 29.5% after 36 months (Figure 1C, merged data sets). Sub‐analyses showed higher rates of *Remission* in comorbid patients: at 36 months, asthmatic patients reached *Remission* in 51.3%, while N‐ERD patients achieved 75.1%. These clear trends, however, lacked statistical significance (*p* = 0.19).

Most importantly for future discussions, *Control* rates above 40% appear high but must not be confounded with treatment *Response*, a term often used in biologic therapy trials [5]. We matched current EUFOREA scores of treatment *Response* upon biologic treatment to our here‐observed diseases states of *Control* and *Remission* (Figure 2). As expected, high scores of 4 points and above correlate well with the likelihood of *Control* (OR 5.52; 95% CI 1.2–25.7); however, roughly 60% of patients without *Control* at 12 months experienced a *good* or *excellent Response* to biologic treatment. Vice versa, 9.5% of patients with *Remission* at 12 months had experienced a *moderate Response* to biologic treatments, making predictions based on the scoring system of treatment *Response* before thorough assessment of the disease state difficult at this moment. Previous literature likewise reported strong responses to Dupilumab, but *Remission* rates varied from 11% to 64% [3, 6], also because of different practical adaptation of recommended criteria.

**FIGURE 2:**
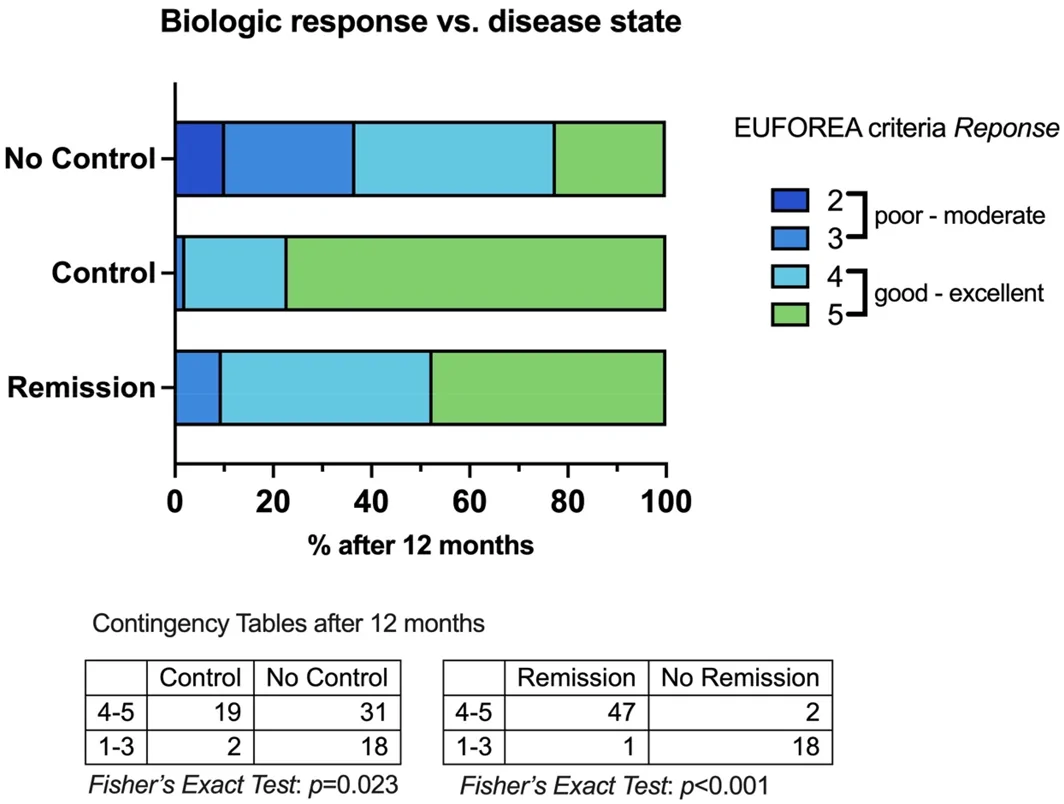
Juxtaposition of disease state (uncontrolled disease, control, *Remission*) at 12 months follow‐up and biologic therapeutic *Response* (based on Fokkens et al.) [5]. Emphasis on the “No Control” bar at the top, that is even‐balanced of patients of all therapy responses. Good and excellent therapeutic *Response* was seen frequently: an average of 4.2 out of 5 criteria had improved after 12 months. 47 patients in *Remission* demonstrated a good—excellent *Response*, whereas 31 patients with good—excellent *Response* did not qualify as controlled. *Response* scores as defined per EUFOREA recommendations were available for *n* = 118.

Discussion of *Control*, *Remission*, and potential therapeutic endpoints will continue to evolve [2]. We identified three main findings that come with current challenges of practical application of the current criteria. One of the main limitations, also in this study, comes with incomplete documentation of strictly VAS‐based symptom severity. Currently, health care systems do not reward achieving *Remission* or *Control*, nor do systemic treatment guidelines allow de‐escalation once *Remission* is reached [7]. If future standards integrate such options, this could influence multimodal therapy concepts including the future position and importance of sinus surgery, including timing. The question of timing is also relevant for the definition of *Remission* and whether a longer period of persistent *Control* must elapse prior to achieving it. In a recent Delphi process, the Italian RINET group discussed such questions of future *Remission* criteria and voted in favor of a 12‐months “waiting” period [8]. Alternatively, mirroring current standards of oncologic principles and recent experiences with biologic therapy, *Remission* could be observed at the next follow‐up visit after completion of any treatment phase independent of its length. In this regard, ensuring continuous *Control* for 12 months with only 2 to 3 follow‐ups is challenging and might miss exacerbations. Under current standards, *Remission* should not be considered possible within the first year, as symptom resolution is not immediate. These questions of therapeutic consequences, timing of *Remission*, grading of *Remission* (partial/complete, as suggested by Heffler et al. [8]) will be among the topics to discuss in future projects. Large‐scale cohort studies are required to refine diagnostic tools, define cut‐offs for symptoms and endoscopy, and establish observation periods for reliable assessment. Furthermore, comorbidity status should be integrated into *Remission* definitions, acknowledging systemic and shared immunological processes beyond the nose and sinuses.

The EUFOREA/EPOS expert panel recommended visual analogue scales (VAS) for overall symptom severity, nasal obstruction, and olfactory impairment. VAS correlates with other scores such as TNSS and SNOT‐22 but not always with psycho‐physical measurement of olfactory function (Supplementary Information S1: Table 1). However, defining cut‐offs for burdensome symptoms remains difficult. Transposing SNOT‐22 items to replace missing VAS data is questionable, as SNOT‐22 measures quality of life, not individual symptoms [3]. We wish to emphasize that the here‐described differences between VAS‐based and adapted criteria‐data sets is not a by‐product to be minimized, but one of the intended findings of this Letter: it demonstrates empirically that heterogeneous, “adapted” operationalization of the same consensus definition produces substantially different *Control* and *Remission* rates.

In conclusion, our multi‐national analysis shows that disease *Control* and *Remission* are increasingly attainable with type‐2 biologics, particularly in patients with asthma and N‐ERD. As these sub‐group differences did not reach statistical significance, they should be regarded as exploratory and hypothesis‐generating; confounding by indication—for example differing baseline severity and closer monitoring in comorbid patients—cannot be excluded. However, current definitions are challenging to apply, and refined, data‐driven standards are needed to guide future therapeutic strategies. Favourable therapy *Response* does not always lead to *Control* and *Remission* as defined by today's standards, which is why more reliable markers for satisfactory patient and disease condition might be out there.

## Supporting information


Supplementry Information S1

## Data Availability

The data that support the findings of this study are available from the corresponding author upon reasonable request.
