# Supplementary material for: Achieving Remission in Chronic Rhinosinusitis With Nasal Polyps Depends on Practical Utilization of Current Criteria and Does not Reflect Therapy Response: Results From a Multi‐National Cohort
Source: Clin Transl Allergy. 2026 Oct 4;16(10):e70211. doi: 10.1002/clt2.70211 (PMC13635686; doi:10.1002/clt2.70211)
Supplement: Supplementary file 1 [file CLT2-16-e70211-s001.docx]

*Online Supplement:*

We addressed heterogeneity of retrospective data collection parameters by using either VAS-based data (VAS nasal obstruction, VAS olfactory impairment <5 from 0 to 10cm) or adapted criteria (VAS global symptoms <5 from 0 to 10cm / SNOT-22<25 points / Sniffin’Sticks 12 item identification testing >8). VAS-based symptom data was available for n=179 cases at 12 months. Whenever VAS-based symptom data was not available, “adapted” criteria for *Control* were put into place in n=190 cases at 12 months (n=57 at 24 months and n=22 at n=36 months). The use of adapted criteria assessing *Control* and *Remission* was leading to higher rates of symptom control, a finding that should be analyzed in further detail in future: most commonly there was a discrepancy between self-reported olfactory function and psychophysical olfactory testing results (12-item *Sniffin’Sticks* identification test), which is a known phenomenon. Whether it is useful to allow/utilize psychophysical, usually more reliable olfactory testing results for the assessment of *Control* should be discussed in future based on larger data sets.

Remission was based on continuous Control for 12 months (0-12 months / 6-24 months / 12-24 months / 24-36 months) and visible absence of active disease by nasal endoscopy. Unilateral nasal polyp score was limited to 1 and total nasal polyp score (NPS) limited to 2. Sinus surgery and/or use of systemic steroids (=rescue treatment) during that particular timeframe ruled out Remission (n=11).)

**Supplemetary Table 1: Data table**

|  | *n* / mean values | | | | |
| --- | --- | --- | --- | --- | --- |
|  | Baseline | 6 months | 12 months | 24 months | 36 months |
| Asthma + | 223 | 223 | 223 | 90 | 63 |
| N-ERD + | 110 | 110 | 110 | 47 | 34 |
| Dupilumab | 274 | 268 | 268 | 115 | 63 |
| Omalizumab | 29 | 29 | 29 | 13 | 5 |
| Mepolizumab | 72 | 72 | 72 | 13 | 4 |
| VAS global symptoms | 7,9 | 3,6 | 3,5 | 1,6 | 1,9 |
| VAS obstruction | 6,8 | 2,0 | 1,8 | 1,6 | 1,2 |
| VAS olf. Impairment | 8,9 | 3,5 | 3,1 | - | - |
| SNOT-22 Q | 52,3 | 24,9 | 23,5 | 15,6 | 16,0 |
| Sniffin'Sticks 12-item | 3,8 | 6,1 | 6,2 | 9,2 | 9,1 |
| Nasal Polyp Score (endosc.) | 5,1 | 2,3 | 1,8 | 1,2 | 0,8 |
| No Control | n/a | 239 | 216 | 85 | 52 |
| Control | n/a | 136 | 153 | 74 | 36 |
| Remission | n/a | n/a | 89 | 32 | 26 |
| Rescue (OCS/surgery) | 11 | | | | |

**Supp. Figure 1**: flow chart depicting reasonst for cohort size reduction.

Further literature:

- Cotter, R.A., et al., *Patient-reported disease control versus overall symptom severity as global metrics of chronic rhinosinusitis disease status.* Int Forum Allergy Rhinol, 2025. **15**(1): p. 27-35.
- Dietz de Loos, D.A.E., et al., *Measuring control of disease in Chronic Rhinosinusitis; assessing the correlation between SinoNasal Outcome Test-22 and Visual Analogue Scale item scores.* Rhinology, 2023. **61**(1): p. 39-46.
- Doulaptsi, M., et al., *Visual analogue scale for sino-nasal symptoms severity correlates with sino-nasal outcome test 22: paving the way for a simple outcome tool of CRS burden.* Clin Transl Allergy, 2018. **8**: p. 32.
- Lötsch, J. and T. Hummel, *Clinical Usefulness of Self-Rated Olfactory Performance-A Data Science-Based Assessment of 6000 Patients.* Chem Senses, 2019. **44**(6): p. 357-364.
- Djupesland PG, Reitsma S, Hopkins C, Sedaghat AR, Peters A, Fokkens WJ. Endoscopic grading systems for nasal polyps: are we comparing apples to oranges? Rhinology. 2022 Jun 1;60(3):169-176. doi: 10.4193/Rhin21.401. PMID: 35403178.
